# Accumulation of Astaxanthin Was Improved by the Nonmotile Cells of* Haematococcus pluvialis*

**DOI:** 10.1155/2019/8101762

**Published:** 2019-02-05

**Authors:** Feng Li, Minggang Cai, Mingwei Lin, Xianghu Huang, Jun Wang, Xuehong Zheng, Shaoting Wu, Yu An

**Affiliations:** ^1^College of Ocean and Earth Science, Xiamen University, Xiamen 361101, China; ^2^Xiamen Ocean Vocational College, Xiamen 361101, China; ^3^College of Fisheries, Guangdong Ocean University, Zhanjiang 524088, China; ^4^Key Laboratory of Marine Chemistry and Applied Technology, Fujian Province, Xiamen 361101, China

## Abstract

The current commercial production of natural astaxanthin is mainly carried out using* Haematococcus pluvialis* vegetative cells in the “two-stage” batch mode. The motile vegetative cells are more sensitive to stress than nonmotile vegetative cells, thereby affecting the overall astaxanthin productivity in* H. pluvialis* cultures. In this study, we compared the differences between motile cells and nonmotile cells in astaxanthin productivity, morphological changes, the mortality rate, and the diameter of the formed cysts. The experimental design was achieved by two different types* H. pluvialis* cell under continuous light of 80 *μ*mol photons m^−2^ s^−1^ for a 9-day induction period. The highest astaxanthin concentration of 48.42 ± 3.13 mg L^−1^ was obtained in the nonmotile cell cultures with the highest the productivity of 5.04 ± 0.15 mg L^−1^ day^−1^, which was significantly higher than that in the motile cell cultures. The microscopic examination of cell morphological showed a large number of photooxidative damaged cells occurring in the motile cell cultures, resulting in higher cell mortality rate (22.2 ± 3.97%) than nonmotile cell cultures (9.6 ± 0.63%). In addition, the analysis results of cell diameter statistics indicated that nonmotile cells were more conducive to the formation of large astaxanthin-rich cysts than motile cells. In conclusion, the works presented here suggest that the accumulation of astaxanthin was significantly improved by nonmotile cells of* H. pluvialis*, which provided a possibility of optimizing the existing* H. pluvialis* cultivation strategy for the industrial production.

## 1. Introduction

Astaxanthin is a high-value red ketocarotenoid with powerful antioxidant capacity [1, 2] and widely used in nutraceuticals, aquaculture, cosmetics, food, and feed industries [3–6]. Because of the high market potential of natural astaxanthin, the efficient production of natural astaxanthin has become one of the main concerns in the industrial production of astaxanthin. The green microalga* Haematococcus pluvialis* is well known as the best source of natural astaxanthin, containing up to 4% of the total cellular dry weight, mainly corresponds to 3S, 3'S isomer, and it is cultivated in industrial scale [7, 8].

The common strategy for production of astaxanthin from* H. pluvialis* in industrial is “two-stage” batch method, consisting of a first step to sustain green vegetable cells rapid growth under favorable conditions (“green” stage) and then a second step carried out by exposing the cells into stress conditions inducing astaxanthin accumulation (“red” stage) [9–12]. At the red stage, the green vegetable cells transformed into red cysts with a thick cell wall by various stress conditions. Light intensity or nutrient depletion was considered as the major factors that stimulate the synthesis of astaxanthin in* H. pluvialis* [13, 14]. It has been reported that high temperature and high salt can also enhance the accumulation of astaxanthin [15–17]. But these stress factors may cause cell death, resulting in the fact that overall astaxanthin productivity in* H. pluvialis *cultures is low [18, 19].

The green vegetative cells of* H. pluvialis* typically include two cell types, motile- and nonmotile cells [20, 21]. The motile cells refer to the swimming cells driven by two flagella, including zoospores which came from asexual reproduction of* H. pluvialis*. The motile cells lost their flagella and developed into spherical nonmotile cells under adverse environment [8]. Most of the previous studies focused on the production of astaxanthin using* H. pluvialis* vegetable cells (the mixture of motile and nonmotile cells) [16, 17, 22, 23]. However, little information was reported on the accumulation of astaxanthin using the nonmotile cell of* H. pluvialis*.

In this study, with the goal of improving the production of astaxanthin by* H. pluvialis*, we mainly examined the astaxanthin accumulation of nonmotile cells of* H. pluvialis* during the induction period. Additionally, the morphology, mortality, and the diameter of red cysts formed were also investigated. The results obtained in this work suggest that nonmotile cells instead of motile cells to stress conditions can significantly improve the production of astaxanthin; this provided an optimized possibility for the existing strategy for the production of astaxanthin from* H. pluvialis*.

## 2. Materials and Methods

### 2.1. Algal Strain and Growth Conditions

The microalgae* H. pluvialis* CCMA-451 was obtained from CCMA (Center for Collections of Marine Algae, Xiamen University, Xiamen, China) and the accession number in the Genbank is MG847145.1. Stock cultures of* H. pluvialis* were maintained at 25 *μ*mol photons m^−2^ s^−1^ in liquid Bold Basal Medium (BBM). Motile cells were grown photoautotrophic in BBM with 0.75 g L^−1^ NaNO_3_ under continuous low light (25 *μ*mol photons m^−2^ s^−1^) for 5 days. For the preparation of nonmotile cells, the vegetative cells from the stock cultures were collected and concentrated by centrifugation (2000 rpm, 2 min) and the supernatant was removed. The collected cells were transferred, in 1-L glass columns (inner diameter 5 cm) containing 600 mL of phosphate-starvation medium, under low light conditions for 5 days. To increase the quantity of nonmotile cells, cells that settled at the bottom of the glass columns were collected and washed with fresh aseptically medium several times to remove remaining motile cells. In the experiments, the cultures of two types of cell (motile and nonmotile cells) were adjusted with induction medium (Table S1) to achieve 0.5 × 10^6^ cell mL^−1^ of cell density and then exposed to continuous light of 80 *μ*mol photons m^−2^ s^−1^ at 25 ± 1°C for 9 days. All the cultures were aerated with 1.5% (v/v) CO_2_ continuously at 0.5 vvm. Illumination was provided from the side by LED plant which grows white lights (Xiamen Top-Succeed Photobiology Technology Co., Ltd., Xiamen, China). Each type of cells was repeated in triplicate.

### 2.2. Morphological Observation

The algal cells were examined using Leica DM750 light microscope and taken photos with Leica ICC50 W camera. Leica application software was used for picture editing.

### 2.3. Determination of Cell Number and Cell Diameter

The samples were fixed with Lugol's iodine solution first, then cell numbers were counted using a Neubauer improved cell counting chamber under Leica DM750 light microscope and measured as cells mL^−1^. The living and dead cells were identified according to the cell morphology shown in Figure S1.

The cell diameter of red cysts was determined using a Leica application software with an internal reticle scale.

### 2.4. Pigment Extraction and Analysis

The astaxanthin concentration was determined photometrically [24, 25]. The samples were collected by centrifugation at 7000 rpm for 5 min. Then the pellet was treated with 5 mL solution of 5% (w/v) KOH in 30% (v/v) methanol in a 75°C water bath for 10 min to remove the chlorophyll. The remaining pellet was then extracted with DMSO after adding 25 *μ*L acetic acid at 75°C for 10 min. This last step was repeated several times to colorless and recover the astaxanthin. The absorbance of the combined extracts was measured at 492 nm (*E*_1  *cm*_^1%^=2220), and the astaxanthin concentration was calculated with

(1) $$\begin{array}{c} C=\frac{{OD}_{492}\times 1000}{E_{1\;cm}^{1\%}\times 100}\times \frac{V_{a}}{V_{b}}\times f \end{array}$$

in which C is the astaxanthin concentration (mg L^−1^), *V*_a_ is the volume of extracted pigment sample (mL), *V*_b_ is the volume of sample (mL), and* f* is the dilution ratio of measuring the absorbance.

The astaxanthin productivity (mg L^−1^ day^−1^) was calculated with

(2) $$\begin{array}{c} \text{Astaxanthin productivity}=\frac{\left({AX}_{t}-{AX}_{0}\right)}{t} \end{array}$$

in which the AX_t_ and AX_0_ were the astaxanthin concentration of day *t* and day 0, respectively.

## 3. Results and Discussion

### 3.1. Astaxanthin Accumulation

**Figure 1:**
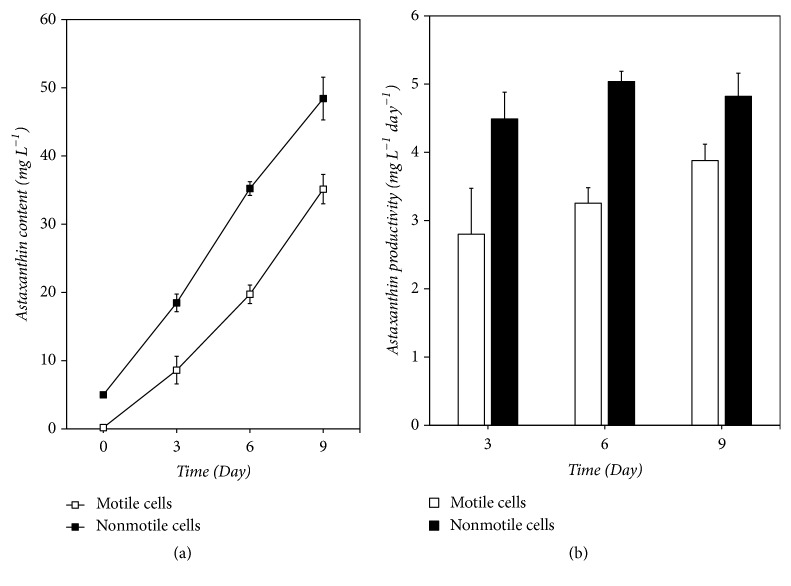
The astaxanthin concentration (a) and productivity (b) of* H. pluvialis* cultures.

The ability of astaxanthin accumulation is the key parameter for evaluating the application potential of algae strains in* H. pluvialis* astaxanthin production. It was reported that astaxanthin synthesis can occur in both motile and nonmotile cells of* H. pluvialis* [20, 21, 26]; however the differences of the astaxanthin accumulation between them still unknown. To compare the differences in astaxanthin accumulation between motile and nonmotile cells, we examined the contents and productivity of astaxanthin in the two cultures. As shown in Figure 1, the nonmotile cell cultures exhibited maximum astaxanthin content (Figure 1(a)). Considering that the initial astaxanthin content in both cultures was different, we furtherly calculated the astaxanthin productivity and the results is shown in Figure 1(b). The value of astaxanthin productivity in the nonmotile cells cultures was ranged from 4.49 ± 0.39 to 5.04 ± 0.15 mg L^−1^ day^−1^ and the maximum value occurred on day 6. It was significantly higher than that of the motile cell cultures. For the motile cell cultures, the value of astaxanthin productivity ranged from 2.80 ± 0.67 to 3.88 ± 0.24 mg L^−1^ day^−1^. The astaxanthin productivity was affected by many factors, such as strains, bioreactors, stress conditions, and initial biomass density in the red stage [8, 11, 16, 27]. A highest astaxanthin productivity of 17.1 mg L^−1^ day^−1^ was obtained at 0.8 g L^−1^ initial biomass density in an outdoor photobioreactor by Wang et al. [27]. In our recent work, the highest astaxanthin productivity in nonmotile cells cultures reached 11.8 mg L^−1^ day^−1^ at 0.5 g L^−1^ initial biomass density under high light conditions (unpublished). Therefore, there was much room for improvement in the production of astaxanthin by nonmotile cells of* H. pluvialis*.

Astaxanthin was regarded as a long-term defense mechanism in* H. pluvialis*, serving as a physicochemical barrier, protecting the cell survival under stress conditions [28, 29]. The astaxanthin accumulation generally was accompanied with the formation of encystment [28, 30–32]. Encystment was also considered as a manifestation of the natural algal defense system [33, 34]. Both astaxanthin accumulation and encystment were the responses of* H. pluvialis* cells to unfavorable conditions; they all needed extra energy consuming. For nonmotile cells, the secondary carotenoids and carbohydrate, which accumulated during the process of transformation, as the precursor and energy provider accelerated the astaxanthin synthesis [35]. On the other hand, due to the high susceptibility to stress conditions [27, 36], cell death occurred largely in motile cells cultures, accordingly the astaxanthin production rate was lower than in nonmotile cells cultures.

### 3.2. Microscopic Examination

**Figure 2:**
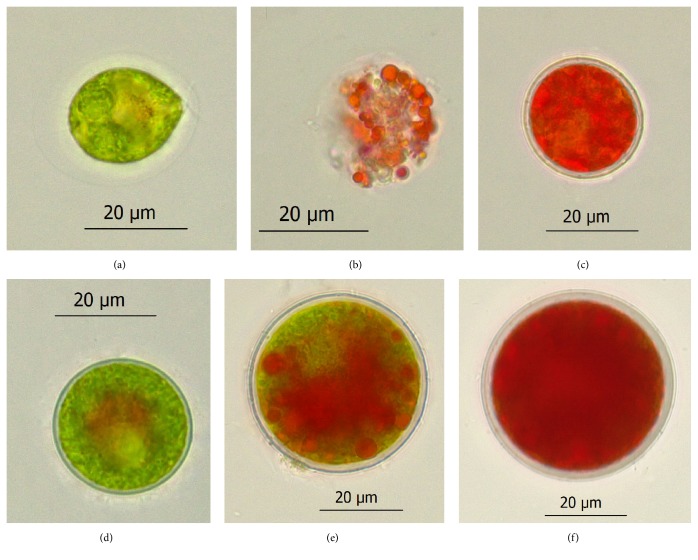
Photomicrographs of* H. pluvialis *cells grown in the red stage. Green motile cell (a) and nonmotile cell (d). The motile cell (b) and nonmotile cell (e) after exposure to phosphate and nitrate starvation at 80 *μ*mol photons m^−2^ s^−1^ for 3 days. The red cyst formed after 9-day induction in the motile cells cultures (c) and nonmotile cells cultures (f).

Changes in cell morphology of motile and nonmotile cells were observed under the light microscope, and the results are shown in Figure 2. The motile cells were green, ellipsoidal, or pear-shaped, with two isometric flagella at the anterior end (Figure 2(a)). Nonmotile cells were spherical, without flagella, little orange-red pigmentation observed in the mid-region (Figure 2(d)). After being induced for 3 days, some motile cells became vacuolated (Figure 2(b)), whereas the nonmotile cells were intact (Figure 2(e)). After being induced for 9 days, almost all of the cells in both of cultures transformed into red cysts with thickened cell walls and accumulated astaxanthin (Figures 2(c) and 2(f)). Moreover, some dead or damaged cells were observed, especially in motile cells cultures.

### 3.3. The Cell Mortality Rate

High light and nutrient depletion were the major environmental factors that promote astaxanthin accumulation [37, 38], but they were able to cause the production of toxic reactive oxygen species (ROS) to result in the cell photooxidative death [39, 40], thereby affecting the overall astaxanthin productivity in* H. pluvialis* cultures. In general, algal cells can dissipate excess light energy and relax ROS production to reduce the photooxidative damage; however it was suggested that these strategies were not sufficient to protect motile cells from environmental stress [23]. In contrast, the nonmotile cells can cope with and survive under stress conditions using several strategies, such as downregulating the linear electron transport through decreasing the level of cytochrome *f* and consuming excess electrons produced by PSII via a significantly enhanced plastid terminal oxidase pathway (POTX) [23]. Furthermore, secondary carotenoids as antioxidants and photoprotective agents, which accumulated during the transformation of nonmotile cells by motile cells, could protect nonmotile cells from environmental stress [33, 41]. Nonmotile cells were also able to effectively convert fixed photochemical energy into storage starch under phosphate-starvation condition and subsequently convert it into storage neutral lipids [23]. In this study, the initial cell mortality rate was 1.0 ± 0.30% and 1.9 ± 0.15% in motile and nonmotile cell cultures, respectively. After induction for 9 days, the cell mortality rate in nonmotile cells cultures reached 9.6 ± 0.63%; it was significantly lower than that in motile cells cultures (22.2 ± 3.97%) (Table 1). It is indicated that the nonmotile cells may have a stronger tolerance to the adverse environment than motile cells. The difference of tolerance to the adverse environment between the two cells may be one of the reasons for the difference in astaxanthin content between the two cultures. In addition, the relatively low mortality of the nonmotile cell cultures may be helpful for enhancing the content of astaxanthin and the stability of astaxanthin production.

### 3.4. The Size of the Red Cysts Formed

**Figure 3:**
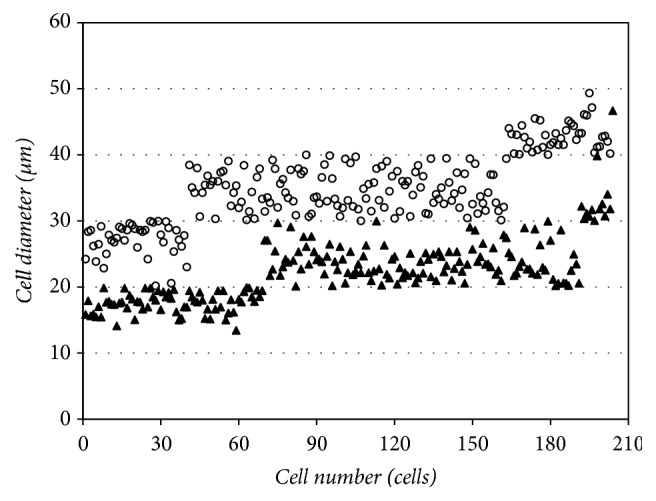
The distribution of* H. pluvialis* cysts in different diameter ranges in the motile cell culture (black triangle) and the nonmotile cell culture (white circle).

The cell size of different types of* H. pluvialis* varies greatly, the range is from 8 to 50 *μ*m [30]. However, the reasons for such a large variation in cell size remain unknown, the correlation between cell type and the size of the red cysts formed are also unclear. In this study, we observed that most of the red cysts in nonmotile cells cultures were bigger than that in motile cells cultures and measured the cell diameter of red cysts in both cultures. As shown in Figure 3 and Table 1, the red cyst diameter in nonmotile cells culture ranged from 20.19 to 49.35*μ*m, the average was 34.82 ± 5.62 *μ*m, whereas, in the motile cell cultures, the diameter of red cyst ranged from 13.45 to 46.69 *μ*m and the average was 22.30 ± 4.74 *μ*m. When environmental or culture conditions became less favorable, motile cells lost their flagella and developed into nonmotile form [22]. Once the culture conditions became unfavorable, cells entered a resting stage to accumulate astaxanthin with the formation of cysts cells (encystment) [25]. Encystment was believed to control by unknown intracellular signaling chains leading to gene expression initiated by environmental signals [42]. Previous studies have demonstrated the formation of* H. pluvialis* encystment accompanied by massive accumulation of carbohydrate, fatty acids, and secondary carotenoids [25, 43]. Thus, it was possible that the enlargement of cells was in order to provide more storage space for these increased compounds.

Cell diameter statistics showed that 33.7% of red cysts in the motile cell cultures were less than 20 *μ*m in cell diameter, 59.5% were in the range of 20 to 30 *μ*m, and 6.3% were in the range of 30 to 40 *μ*m, while the values of the same range in nonmotile cells cultures were 0%, 19.7%, and 60.6%, respectively. In addition, about 19.7% of red cysts in the nonmotile cells cultures were bigger than 40 *μ*m, while the cell percentage of this range in motile cells cultures was only 0.5% (Figure 3). We furtherly calculated the astaxanthin content of single red cyst in both cultures; the results showed that the average astaxanthin content of a single red cyst in the nonmotile cell cultures was 1.94 times as high as that in the motile cell cultures (Table 1).

These results supported the fact that the nonmotile-type cells can form larger red cysts and accumulate higher astaxanthin than motile-type cells, which suggest that increasing the number of nonmotile-type cells has a positive effect on improving the astaxanthin content of* H. pluvialis*.

## 4. Conclusions

In this study, we have determined the fact that the accumulation of astaxanthin is significantly improved by the nonmotile cells of* H. pluvialis* compared with motile cells. We show that nonmotile cells have higher astaxanthin productivity than motile cells. The results of the cell mortality rate indicate that the nonmotile cells may have a stronger tolerance to the adverse environment than motile cells. In addition, the analysis results of cell diameter statistics indicate that nonmotile cells are more conducive to the formation of large astaxanthin-rich cysts than motile cells. These results may account for the improvement of astaxanthin accumulation in nonmotile cell cultures. Our work presented here provides a possibility of optimizing the existing cultivation strategy by the nonmotile cells as the primary cell type to improve the stability and overall astaxanthin productivity in industrial cultivation of* H. pluvialis*.

## Figures and Tables

**Table 1:** The average cyst diameter, astaxanthin content, and cell mortality rate in motile and nonmotile cell cultures on day 9 of the induction period.

Cultures	Average cyst diameter (*μ*m)	Astaxanthin content (pg cell^−1^)	The cell mortality rate (%)
Motile cell cultures	22.30 ± 4.74	22.79 ± 0.75	22.20 ± 3.97
Nonmotile cell cultures	34.82 ± 5.62	44.30 ± 4.47	9.60 ± 0.63

## Data Availability

No data were used to support this study.

## Supplementary Materials

Supplementary Materials Table S1: chemical compositions of induction culture media for H. pluvialis used in this study. Figure S1: the cell morphology of living and dead cells of H. pluvialis.
