# Supplementary material for: Accumulation of Astaxanthin Was Improved by the Nonmotile Cells of Haematococcus pluvialis
Source: Biomed Res Int. 2019 Feb 5;2019:8101762. doi: 10.1155/2019/8101762 (PMC6379868; doi:10.1155/2019/8101762)
Supplement: Supplementary Materials — Table S1: chemical compositions of induction culture media for H. pluvialis used in this study. Figure S1: the cell morphology of living and dead cells of H. pluvialis. [file 8101762.f1.zip › Support Material.docx]

**Supplementary Materials**

[Table S1. Induction medium for *H. pluvialis* 1](#_Toc534295086)

[Figure S1. The cell morphology of living and dead cells of *H. pluvialis* 2](#_Toc534295087)

# Table S1. Induction medium for *H. pluvialis*

| Stock Solution (SL) | Volume | Component | Concentration in SL | Concentration in final Medium |
| --- | --- | --- | --- | --- |
| SL 1 | 10 mL | MgSO_4_.7H2O | 7.5 g L^-1^ | 3.04×10^-4^ M |
| SL 2 | 10 mL | NaCl | 2.5 g L^-1^ | 4.28×10^-4^ M |
| SL 3 | 10 mL | CaCl_2_.2H_2_O | 2.5 g L^-1^ | 1.70×10^-4^ M |
| SL 4 | 1mL | ZnSO_4_.7H_2_O | 8.82 g L^-1^ | 3.07×10^-5^ M |
|  |  | MnCl_2_.4H_2_O | 1.44 g L^-1^ | 7.28×10^-6^ M |
|  |  | MoO_3_ | 0.71 g L^-1^ | 4.93×10^-6^ M |
|  |  | CuSO_4_.5H_2_O | 1.57 g L^-1^ | 6.29×10^-6^ M |
|  |  | Co(NO_3_)_2_.6H_2_O | 0.49 g L^-1^ | 1.68×10^-6^ M |
| SL 5 | 1mL | H_3_BO_3_ | 11.4 g L^-1^ | 1.85×10^-4^ M |
| SL 6 | 1mL | EDTA.Na_2_ | 50 g L^-1^ | 1.71×10^-4^ M |
|  |  | KOH | 31 g L^-1^ | 5.53×10^-4^ M |
| SL 7 | 1mL | FeSO_4_.7H_2_O | 4.98 g L^-1^ | 1.79×10^-5^ M |
|  |  | H_2_SO_4_ | 1 mL |  |

For 1000 mL final culture medium add the quantities (Volume) of stock solutions (SL) prepared at the given concentrations in Table S1 to 870 mL deionized water. Add one component after the other until each one has completely mixed and finally fill up to 1000 mL. All stock solutions can be stored unsterilized at 4 °C.


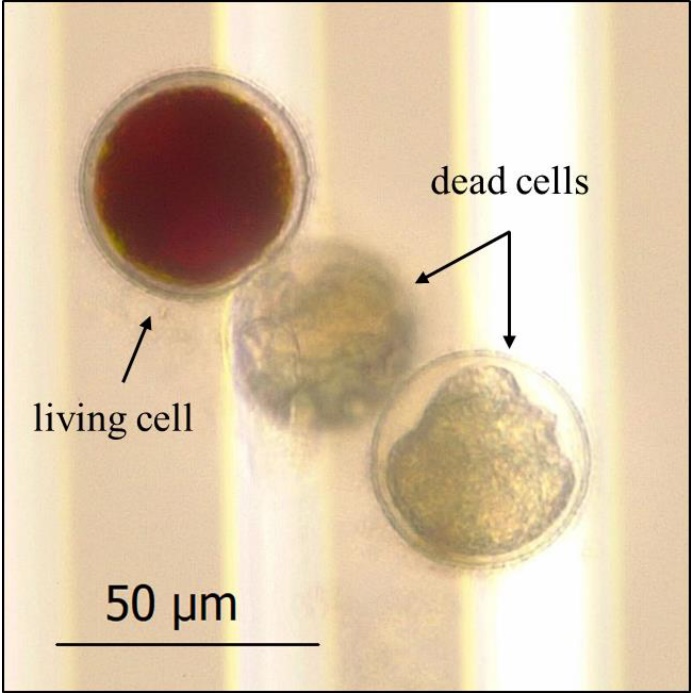


# Figure S1. The cell morphology of living and dead cells of *H. pluvialis*
