# Supplementary figures and images for: Accumulation of Astaxanthin Was Improved by the Nonmotile Cells of Haematococcus pluvialis
Source: Biomed Res Int. 2019 Feb 5;2019:8101762. doi: 10.1155/2019/8101762 (PMC6379868; doi:10.1155/2019/8101762)

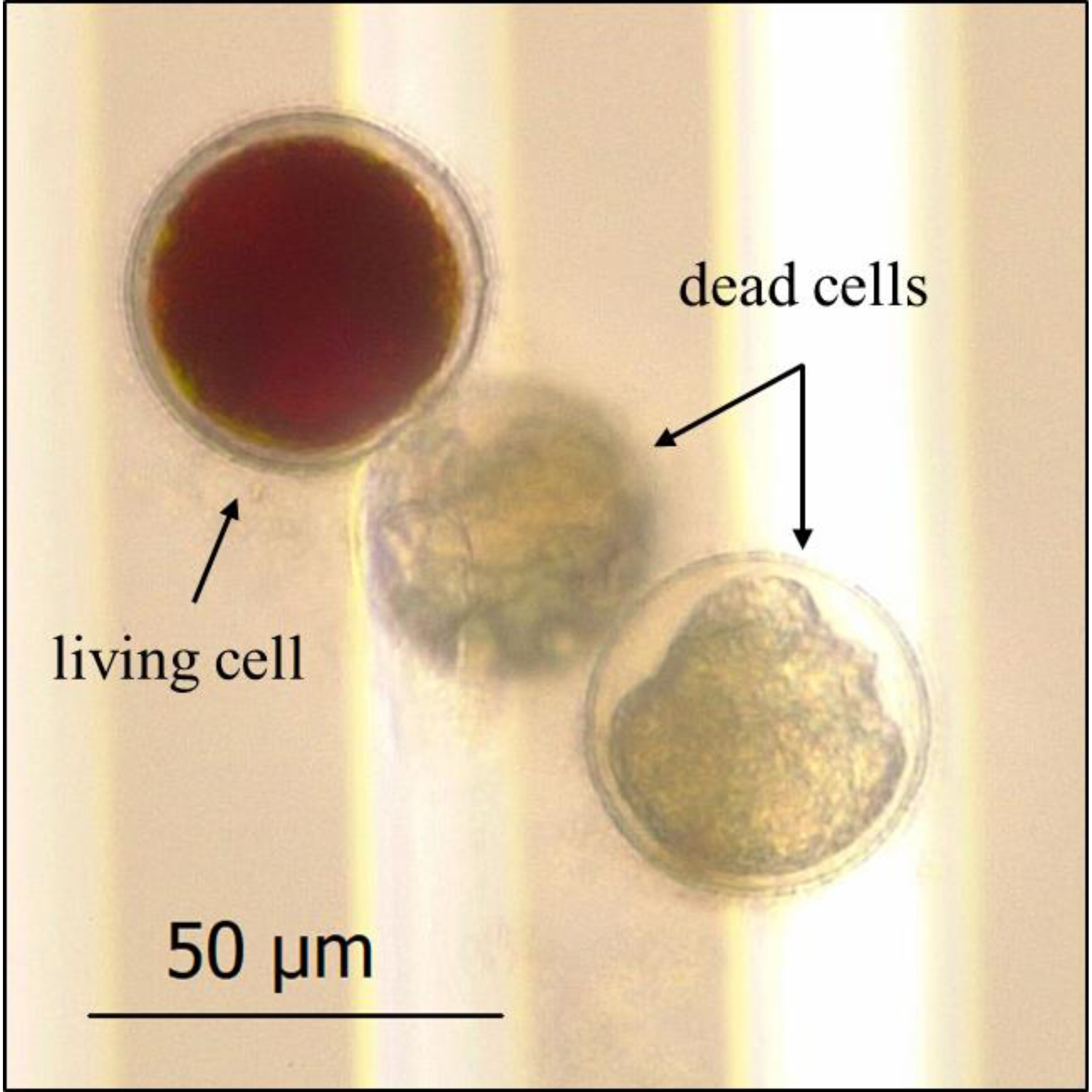

Supplement: Supplementary Materials — Table S1: chemical compositions of induction culture media for H. pluvialis used in this study. Figure S1: the cell morphology of living and dead cells of H. pluvialis. [file 8101762.f1.zip › Figure S1.tif]
